# Towards a definition of refractory neuropathic pain for epidemiological research. An international Delphi survey of experts

**DOI:** 10.1186/1471-2377-12-29

**Published:** 2012-05-28

**Authors:** Blair H Smith, Nicola Torrance, Janice A Ferguson, Michael I Bennett, Michael G Serpell, Kate M Dunn

**Affiliations:** 1 Medical Research Institute, Mackenzie Building, Kirsty Semple Way, University of Dundee, Dundee, DD2 4BF, UK; 2 Leeds Institute of Health Sciences International, University of Leeds, Leeds, UK; 3 Gartnavel General Hospital, University Department of Anaesthesia and Pain Management, Glasgow, UK; 4 Arthritis Research UK Primary Care Centre, Keele University, Keele, Staffordshire, UK

**Keywords:** Neuropathic pain, Refractory, Epidemiology, Delphi method, Web-based questionnaire

## Abstract

**Background:**

Best current estimates of neuropathic pain (NeuP) prevalence come from studies using various screening detecting pain with probable neuropathic features; the proportion experiencing significant, long-term NeuP, and the proportion not responding to standard treatment are unknown. These “refractory” cases are the most clinically important to detect, being the most severe, requiring specialist treatment.

**Methods:**

We report an international Delphi survey of experts in NeuP, aiming for consensus on the features required to define, for epidemiological research: (1) neuropathic pain; and (2) when NeuP is “refractory”. A web-based questionnaire was developed and data collected from three rounds of questionnaires from nineteen experts.

**Results:**

There was good consensus on essential inclusion of six items to identify NeuP (“prickling, tingling, pins & needles”, “pain evoked by light touch”, “electric shocks or shooting pain”, “hot or burning” pain, “brush allodynia on self-examination”, and “relevant history”) and on some items that were non-essential. Consensus was also reached on components of a “refractory NeuP” definition: minimum duration (one year); number of trials of drugs of known effectiveness (four); adequate duration of these trials (three months / maximum tolerated); outcomes of treatment (pain severity, quality of life). Further work needs to validate these proposed criteria in general population research.

**Conclusions:**

This paper presents an international consensus on measuring the epidemiology of refractory neuropathic pain. This will be valuable in reaching an agreed estimate of the prevalence of neuropathic pain, and the first estimate of refractory neuropathic pain prevalence.

## Background

Studies have examined the prevalence, distribution and determinants of specific conditions associated with neuropathic pain (NeuP), notably postherpetic neuralgia (PHN) and painful diabetic neuropathy (PDN) [1]. However, the overall population prevalence of NeuP remains unknown, and factors associated with its onset and recovery are poorly understood. Prevalence estimates of 1–2% [2,3] are inaccurate (as they are extrapolated from assumptions relating to specific conditions) and are probably under-estimates [4], as they do not include undiagnosed NeuP or cases where neuropathic features contribute importantly to pain of mixed mechanism [5].

The current best population estimates for NeuP come from studies using screening instruments that detect pain with probable neuropathic features. One of these, using the S-LANSS, found a prevalence of “pain of predominantly neuropathic origin” (POPNO) of 8.2% [6]. Another, using the DN4, found a prevalence of “chronic pain with neuropathic characteristics” of 6.8% [7]. Using the PainDETECT questionnaire, Freynhagen et al [8] estimated that 11.4% of males had low back pain with a predominantly neuropathic component. Differences in these and other estimates are partly accounted for by differences in the way in which NeuP is defined by the different instruments, which were originally defined for clinical purposes [9]. Whilst there is no ideal epidemiological method for defining and ascertaining NeuP, it is clear that specialist examination is impractical in large population studies, and other methods, such as reviewing medical records, are reliant on the quality and standard criteria of routine data entry. There is therefore a need for consensus on a definition of NeuP for use in population research.

### Neuropathic pain that is refractory to treatment

Among these relatively high reported prevalences, it is unknown what proportion of individuals experience clinically significant, long-term pain and/or pain that has not responded to standard non-specialist treatment. These “refractory” cases of NeuP are clinically the most important to detect, as they are likely to be the most severe and difficult to treat, use healthcare services more often, and to be those who merit informed treatment and follow-up targeted at NeuP. The term “refractory neuropathic pain” has emerged recently in the literature, [10-12] but definitions vary markedly. In a preliminary attempt to review the epidemiology of refractory NeuP, Taylor [12] used a search strategy with a broad definition that included specific named NeuP conditions and pain that was *“*persistent*”.* The Scottish Medicines Consortium (SMC) describes patients with refractory NeuP as those who *“*have not achieved adequate pain relief from, or have not tolerated, conventional first and second line treatments for neuropathic pain*”*, with specific prescribing implications [13]. In one randomised controlled trial [11], refractory NeuP was defined as having had lasted at least six months, with a pain severity score of at least 40 mm on a 0-100 mm visual analogue scale, and exhibiting no response to usual pharmacological care. There is, however, neither consensus on these definitions, nor critical review of their parameters.

Hansson *et al.* recently proposed a definition of “pharmacoresistant” NeuP and a rational approach to prescribing efficacious drugs in order of precedence [14]. However, the authors concluded that the required scientific evidence to confirm this definition is not yet available. Furthermore, “refractory NeuP” and “pharmacoresistant NeuP” may be clinically different entities. Attempts to distinguish between refractory and pharmacoresistant epilepsy have proved challenging [15,16], with no final consensus.

If refractory NeuP can be defined and classified in a way that is agreed to be clinically and epidemiologically relevant, it will be possible to identify individuals and sub-groups within the community who experience this most severe NeuP and are in greatest need of treatment. This will in turn allow an assessment of the scale of the problem, identification of risk factors for “refractoriness” (including those that are potentially modifiable), and the subsequent efficient targeting of management or prevention strategies.

The aims of this study were therefore to reach expert consensus on the features required to define, for epidemiological research: (1) neuropathic pain; and (2) when neuropathic pain is “refractory”. These features should be able to be incorporated into a questionnaire suitable for administration to large study samples.

## Methods

### Consensus of experts/the Delphi method

The Delphi technique has been used widely in medical and nursing research as a survey method used to gain consensus among a group of respondents [17-19]. The technique involves asking a panel of experts to take part in a series of consecutive rounds of questionnaires designed to achieve increasing consensus of opinion. A panel usually consists of 15 to 30 participants [18], between 12 and 20 being considered optimal [18,20]. Typically three rounds of questionnaires are sent to the expert panel, although the decision over the number of rounds is largely pragmatic and often varies between two and four partly depending on the quality and rates of response [20-22]. Participants’ responses are anonymised to ensure that the influence of peer pressure on respondents’ opinions is minimised [23]. A summary of the results showing the distribution of the group’s response and patterns of agreement of the previous round are fed back to be evaluated by panel members [19,23]. Consensus can be said to be achieved when a given proportion of participants are in agreement. This proportion varies between studies, with some authors accepting 51% [24] whilst other have suggested levels of between 60% and 80% are required [17,25-27].

#### Participants

Internationally recognised experts on neuropathic pain research were identified. These included authors of important published epidemiological and clinical studies, and members of the Committee of the Neuropathic Pain Special Interest Group (NeuP SIG) of the International Association for the Study of Pain, who developed guidelines on the assessment of neuropathic pain [28]. These individuals were approached by email with an invitation to participate in the Delphi method survey. The final list included 40 experts from 11 countries. To comply with the conditions of ethical approval, the identities of experts were kept confidential throughout the study and beyond. We set *a priori* the level of agreement between them at which consensus would be recognized as 70%, recognizing that more than a simple majority would be required but that the complexity of the concepts involved meant that greater agreement might be unfeasible, or exclude a meaningful definition. Similarly, less than 30% agreement was considered to represent consensus about excluding items.

#### First round

A web-based questionnaire was developed using SurveyMonkey (http://www.surveymonkey.com ). Each expert was sent an email inviting them to take part in the study and a link to access the questionnaire on the SurveyMonkey website.

In this round, we asked about the usefulness of items found in published screening tools developed to identify pain with neuropathic characteristics [29], and which of these individual items *“should be included in an instrument to be utilised in epidemiological research”.* Participants were to choose whether items were essential to include, not essential to include, or whether items should not be included. They were also asked to specify any other essential items.

A further free text question asked participants “*In your opinion, when does neuropathic pain become “refractory”? Please list the crucial features that you consider would categorise a patient as having “refractory neuropathic pain”.*

#### Second round

The results of round one were fed back to each of the participants by email, along with a link to the round 2 questionnaire. This questionnaire asked participants to consider their agreement about the inclusion of all of the items that were included in round 1. Content analysis of the free text responses to the question on the features that characterise refractory NeuP was conducted to identify common attributes to be included in a possible definition. The attributes that were identified were: *“Minimum duration of neuropathic pain”, “Number of drugs of known effectiveness for neuropathic pain tried”, “What might comprise an adequate trial for neuropathic pain treatment?”, “Outcome of treatment” and “Usefulness of other non-drug therapies”. S*pecific levels within each of these attributes were also generated from the free text responses (shown in Table 1).

**Table 1:** Attributes and levels identified for consideration in determining refractoriness of neuropathic pain, and subsequent consensus among participants

	Median (/5)*	Mean*	**Level of consensus,% (n)**	**Final consensus on inclusion/exclusion (>70%)**
**Minimum duration of NeuP**				
Three months	4.00	4.05	**89.5% (17) disagree/strongly disagree**	**YES**
Six months	3.00	2.89	36.8% (7) strongly agree/agree	NO
More than one year	2.00	2.53	52.6% (10) strongly agree/agree	NO
Duration irrelevant	3.00	3.05	42.1% (8) strongly agree/agree	NO
**Minimum number of drugs of known effectiveness for NeuP tried**				
**Two drugs**	**4.00**	**3.83**	**72.2% (13) disagree/strongly disagree**	**YES**
Three drugs	2.00	2.44	66.7% (12) strongly agree/agree	NO
**Four drugs**	**2.00**	1.78	**88.9% (16) strongly agree/agree**	**YES**
**More than four drugs**	**1.00**	1.39	**94.5% (17) strongly agree/agree**	**YES**
**An adequate trial of NeuP treatment**				
One week	5.00	4.78	100% (18) **disagree/strongly disagree**	YES
One month	3.00	3.17	31.6% (6) strongly agree/agree	NO
**Three months**	**2.00**	**2.06**	**83.5% (15) strongly agree/agree**	**YES**
**Until adverse effects prevent adequate dosage**	**1.00**	**1.63**	**89.5% (17) strongly agree/agree**	**YES**
**Outcomes of pain treatment**				
**Pain levels of 5 or more on a 0–10 pain scale**	**2.00**	**2.61**	**83.3% (15) strongly agree/agree**	**YES**
**Less than 30% pain intensity reduction**	**2.00**	**2.06**	**88.9% (16) strongly agree/agree**	**YES**
**No period of pain remission**	**2.00**	**2.12**	**82.4% (14) strongly agree/agree**	**YES**
**An increase in pain severity**	**2.00**	**1.78**	**77.8% (14) strongly agree/agree**	**YES**
**Quality of life remains significantly affected by pain (e.g. sleep, activity limitations, mood, disability)**	**2.00**	**1.83**	**83.3% (15) strongly agree/agree**	**YES**
**Usefulness of other non-drug therapies**				
Non-pharmacological therapies must be tried before neuropathic pain is considered refractory (e.g. TNS, physical therapy, psychological therapy, relaxation)	2.00	2.58	63.2% (12) strongly agree/agree	NO
**No response to spinal cord stimulator**	**4.00**	**3.89**	**73.7% (14) disagree/strongly disagree**	**YES**

*1 = strongly agree to 5 = strongly disagree, lower mean/ median score = higher agreement.

Agreement was sought (from 1 = strongly agree to 5 = strongly disagree) on whether individual items should be included in the definition of neuropathic pain, and for each of the attributes that might determine when this was refractory.

Finally, we asked about any perceived difference between the terms “Refractory Neuropathic Pain” and “Pharmacoresistant Neuropathic Pain”, and whether participants had any preferred terms or further comments about the study.

#### Third round

Individual and group responses were fed back to each participant and they were asked for a final time to indicate their level of agreement with each of the components to be incorporated into a working definition of refractory neuropathic pain.

### Ethical approval

This study was approved by Ethics Review Board of the College of Life Sciences and Medicine, University of Aberdeen.

## Results

**Figure 1:**
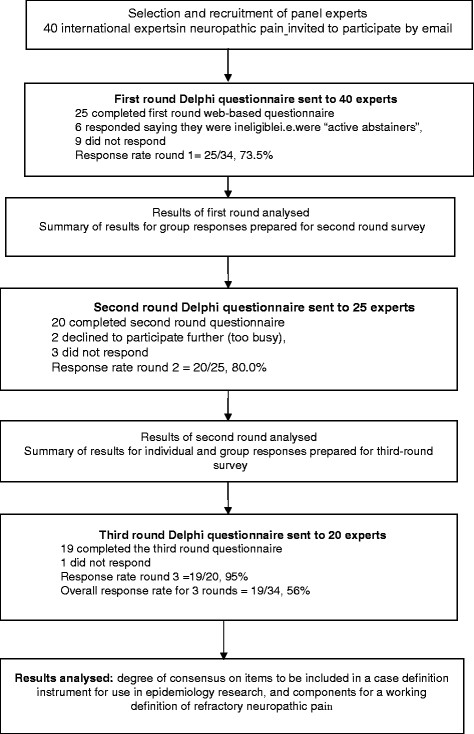
Flowchart of participants in the study.

Of the forty invited experts, six indicated that they were not eligible for the study. Twenty-five of the other experts (73.5%) completed the Round 1 questionnaire. Response rates throughout each round of the study are shown in Figure 1. In total nineteen experts returned completed questionnaires for all three rounds of the Delphi survey. No further participants were sought as the sample had achieved an “optimal size” for a Delphi study [18,20]. Participants in round one (n = 25) represented a range of medical and research disciplines (including neurology, epidemiology, anaesthetics, pain medicine, and neuropharmacology) and were based in nine countries (thirteen were from the UK, seven participants were from five other European countries, four were from North America and one from Australasia).

### Items to be included in the definition of neuropathic pain

**Figure 2:**
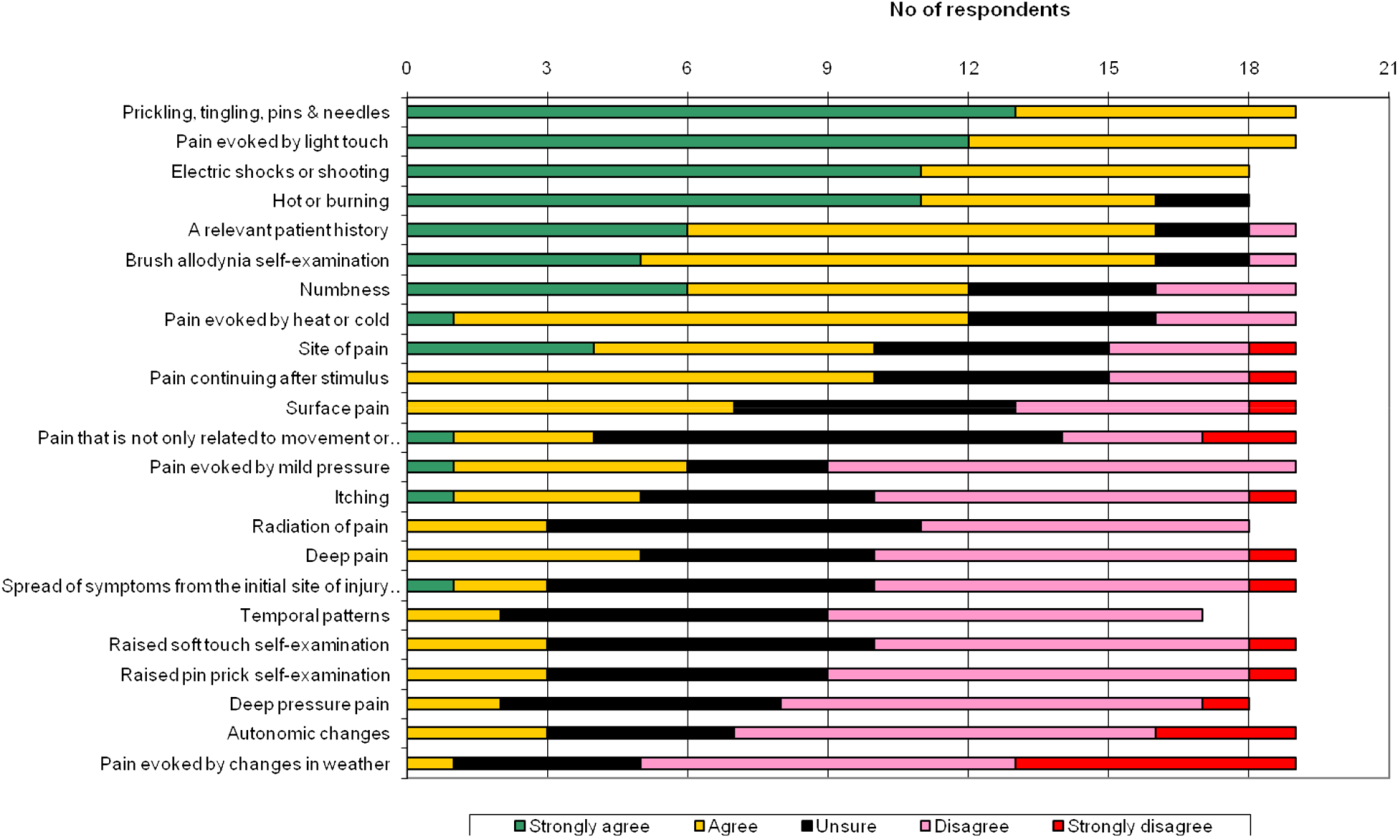
Agreement after three rounds with items about pain characteristics that should be included in a neuropathic pain case definition tool for epidemiological use.

The level of agreement after three rounds for items to be included in a case definition instrument for neuropathic pain in epidemiology research is shown in Figure 2. There was consensus (with at least 70% of participants agreeing or strongly agreeing) that six items about the presence of pain with the following characteristics should be included: “prickling, tingling, pins & needles”, “pain evoked by light touch”, “electric shocks or shooting pain”, “hot or burning” pain, “a relevant patient history”, and “brush allodynia on self-examination”. There was consensus (less than 30% agreeing or strongly agreeing) that eleven items should not necessarily be included. A further six items produced mixed responses and considerable uncertainty about their inclusion (Table 2).

**Table 2:** Case definition items to be included in a questionnaire instrument for epidemiology research

Questions about pain characteristics:
Prickling, tingling, pins and needles
Pain evoked by light touch
Electric shocks or shooting pain
Hot or burning pain
Brush allodynia on self examination
Question eliciting a relevant patient history
*Items that might be included (>30% but <70% consensus)*
Questions about pain characteristics:
Numbness
Pain evoked by heat or cold
Pain continuing after stimulus
Surface pain
Pain evoked by mild pressure
Question about the site of pain

### Definitions of refractory neuropathic pain

All participants in Round 1 completed the question about when neuropathic pain becomes “refractory neuropathic pain”. Some examples of the free text responses are shown in Table 3. Content analysis of all of the responses enabled attributes and levels to be identified for incorporation into the Round 2 questionnaire (see above).

**Table 3:** A selection of free text responses about a definition of refractory neuropathic pain

*“Pain that cannot be reduced to levels of 4 or less on a 1–10 scale after all available biomedical treatments have been given and adequate try”*
*“Does not respond to 3 different classes of neuropathic pain drugs (TCA,> gabapentin/ pregabalin/ opioid at established sufficient dose each for a sufficient time”*
*“Duration > 6 months and unresponsive or poorly responsive to gabapentin/ pregabalin, TCA, topical lidocaine patch, opioid”*
*“Persistent clinical relevant pain despite a proper trial of gabapentin or pregabalin and a tricyclic antidepressant or a serotonin noradrenalin reuptake inhibitor”*
*“When, after appropriate assessment and treatment, patients are still often distressed and/or have activity limitations due to their NP, and also have persistent symptoms of the type outlined above”*
*“Persists more than a year after original injury or lesion. Pain levels greater than 5/10 despite adequate trial with standard class 1 or 2 drugs. Pain significantly affects the quality of life, sleep and daily function despite adequate therapy with pharmacological agents, physical therapy and CAM therapies”*
*“Patients got insufficient pain relief after trying Pregabalin up to 450 mg/die, amitriptyline 1 mg per kilo, oxycodone 20 mg, or could not reach the target dosage because of adverse events. If patients have contraindications to a TCA antidepressant they should try duloxetine 60 mg or venlafaxine 225 mg. If patients have allodynia in a small area they should also try a lidocaine patch. If patients have Trigeminal neuralgia they should try none of the above; they should try oxcarbazepine up to 1600 mg or carbamazepine up to 1000 mg. If they can reach adequate dosage with either drug and still get insufficient pain relief they should be proposed surgery. Concerning the problem of combination therapy, I do not think we have enough evidence to ask for it. Perhaps it may be considered when adverse events prevent reaching the adequate dosage?”*
*“6 months history not responded to first line treatments (ie medication with lidocaine, anticonvulsants, antidepressants) significant disability and distress”*
*“All of the neuropathic pain beyond 3 months is chronic and all of the neuropathic pain that persists at level of 5/10 or higher even with a single or multiple treatment modalities is refractory”*
*“Resistant to any kind of pharmacological and nonpharmacological therapy, if the drugs and doses have been tested along the common guidelines (long and high enough)”*

#### Minimum duration of neuropathic pain

**Figure 3:**
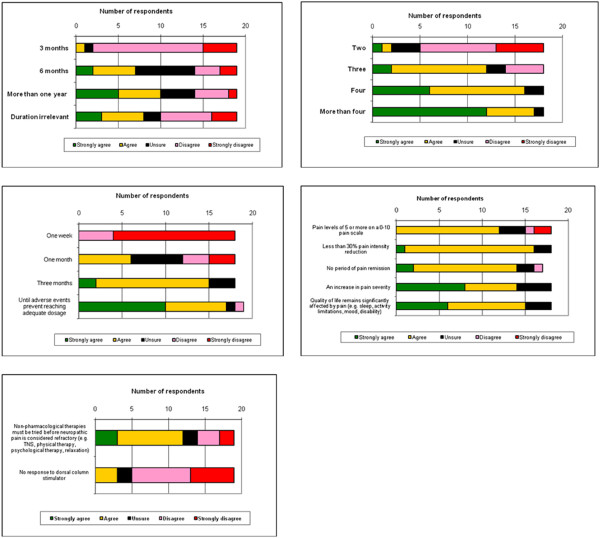
Agreement with levels of attributes identified for a definition of refractory neuropathic pain for use in epidemiology research.

There was no clear agreement on the minimum duration of NeuP for it to become refractory (Table 1, Figure 3a). However, there was clear consensus that three months was an insufficient period of time, as 17/19 (89.5%) of the experts disagreed or strongly disagreed that NeuP could be said to be refractory after this length of time. Strongest agreement was for greater than one year, but many (8/19, 42.1%) considered that duration was not relevant to determining refractory neuropathic pain (Table 1, Figure 3a).

#### Number of drugs of known effectiveness for neuropathic pain tried

There was good agreement that four (16/18, 89% of participants strongly agreed/ agreed), or more than four drugs (17/18, 94.5% of participants strongly agreed/ agreed) of known effectiveness for neuropathic pain had to be tried before it became “refractory” (Table 1 / Figure 3b). Two thirds of participants indicated that a trial of three neuropathic pain drugs was sufficient, and there was consensus (13/18, 72.2% disagreed/strongly disagreed) that two drugs were insufficient.

#### An adequate trial of neuropathic pain treatment

There was consensus that at least three months’ trial of each neuropathic pain treatment was necessary (15/18, 83.3% of participants), or treatment until adverse effects prevented adequate dosage (17/19, 89.4% of participants) before it could be considered refractory.

#### Outcome of treatment

There was consensus that any of the listed outcomes of treatment was sufficient to render neuropathic pain refractory: persistence of pain, failure to reduce pain intensity or severity significantly, an increase in pain severity, or persistently reduced quality of life (Figure 3d).

#### Usefulness of other non-drug therapies

Although 12/19 (63%) participants agreed that non-pharmaceutical therapies (included therapies such as transcutaneous nerve stimulation, physical therapy, psychological therapy, relaxation) should be tried before neuropathic pain is considered refractory, this response did not meet the consensus level (of 70%). There was, though, consensus (14/19, 73.7%) that trial of spinal cord stimulation was not required before NeuP could be considered refractory.

#### Terminology

Sixteen participants (84.2%) agreed or strongly agreed that there was a difference between the terms “Refractory Neuropathic Pain” and “Pharmacoresistant Neuropathic Pain”, with many comments suggesting that both terms were important in their own right.

## Discussion

This study aimed to achieve consensus from an international group of experts in neuropathic pain epidemiology on (1) the components of a questionnaire instrument for identifying neuropathic pain in epidemiology research, and (2) when this pain could be termed “refractory”. Lack of consensus in the first of these has hindered epidemiology research to date, and contributed to the considerable variance in published prevalence rates. This was not intended to supplant the clinical definition of (possible) NeuP [30], but to allow this to be partly operationalised in population-based research. There has previously been no consensus on refractoriness, despite several publications purporting to report on refractory neuropathic pain.

### Identifying neuropathic pain

In summary, we found that a case definition instrument for neuropathic pain should certainly include five questions about pain characteristics, and one eliciting a clinical history consistent with neuropathic pain (Table 2). An additional six items produced mixed responses from the expert group. Given the need for brevity and focus in most population research questionnaires, we propose that only the six items with >70% consensus be included in a standard case definition instrument for epidemiology research. The five characteristics of pain quality identified by survey respondents are all featured within the three most widely used screening tools for neuropathic pain (S-LANSS, DN4, painDETECT) [9,31]. The remaining characteristic (a relevant patient history) does not feature in these screening tools but is part of the diagnostic algorithm advocated by Treede et al [30].

We examined these five characteristics of pain quality against the cut-off scores within each of the screening tools to determine which combination(s) might be used as a case definition tool for epidemiological use:

· DN4: cut-off is score of 4, equivalent to any four items [32].

· S-LANSS: cut-off is score of 12 or more. Of the five possible combinations using four items each, three combinations reach a positive score. The remaining two combinations score 11 [29].

· painDETECT: cut-off score is 13 or more. If three items are rated at least 3 (strongly) and one item 4 (very strongly), a positive score is reached [8].

Based on this analysis, we propose to test the validity of a case definition tool for epidemiological use using any four characteristics of pain quality in combination with questions eliciting ‘a relevant patient history’. It is not proposed that this tool would replace detailed history and examination, which must remain as cornerstones in the clinical setting.

### Determining refractory neuropathic pain

For neuropathic pain to be considered refractory, based on the consensus reported above, we propose that:

· It should have had a trial of treatment with at least four drugs of known effectiveness in neuropathic pain

· Each of these drugs should have been tried for at least three months or until adverse effects prevent adequate dosage

· Despite the above treatment, the intensity of pain should have reduced by less than 30%, or should remain at a level of at least 5 on a 0–10 scale; and/or it should continue to contribute significantly to poor quality of life.

We propose that the duration of neuropathic pain, by itself does, not determine whether it is refractory (though it is clearly relevant when considering the duration of any trials of treatment). We also propose that, while non-pharmacological treatments should be considered, and may be effective, neuropathic pain can be considered refractory even in their absence.

### Strengths and limitations of the study

Delphi surveys are an established method of achieving consensus in complex areas and/or where consensus has not previously been able to be reached [18]. For example previous studies have used Delphi methods to develop a standard definition for back pain for use in prevalence studies (a consensus of 28 experts in back pain research from 12 countries) [33], and to develop guidelines for the management of hip and knee osteoarthritis (a consensus of 16 experts from four medical disciplines and six countries) [27]. The composition of the expert panel is important, and should comprise a reasonable number of individuals, representing a widespread range of experts in the relevant discipline(s).

The response rate to this international study was good, with participation from 25 (73.5% of eligible) invited experts in NeuP in round one, and from 19 (76%) of these experts participating in all three rounds. These included participants from a range of clinical and research disciplines and a number of countries worldwide, all of whom had been previously recognized through international peer selection as experts in their fields. Considerable weight can therefore be given to the items on which clear consensus was achieved. Although the nature of work in this area meant that most of the respondents knew members of the research team, it is unlikely that this would have contributed importantly to any bias in the responses: responses were anonymous, and there appeared to be no hesitation in expressing forthright views. It would have been possible to include more experts, increasing the number of responses and their sources. We were concerned to include individuals who had contributed importantly to the literature in this field, and our sample represents a good selection of these, but it is possible that including experts based in other countries, including the developing world, would have further enriched our responses.

It must be noted, however, that unanimity was not achieved for any item, and there must still be room for flexibility in designing and interpreting questionnaire aiming to identify refractory neuropathic pain. For example, whilst we have proposed that an adequate trial of at least four drugs is required before NeuP should be considered “refractory”, good consensus was nearly achieved (66.7%) on a trial of three drugs. Indeed, two of the experts approached stated their view that it was inadvisable to try to identify neuropathic pain through a questionnaire, preferring instead a clinical assessment. We recognise the distinction between diagnosing neuropathic pain using clinical assessment in order to initiate treatment, and estimating the likelihood of neuropathic pain in a population using proxy criteria in order to understand epidemiological factors. Whilst we respect the importance of rigorous clinical examination in providing a detailed assessment of neuropathic pain, we maintain that this is not practical for research in studies of the sample size required for accurate epidemiology. We are not proposing the above case definition questionnaire as a “gold standard” for neuropathic pain, but as a rigorous consensus, for research purposes only, which may now be tested in the field. Clinical examination is required to identify and assess “definite” neuropathic pain [30], (though this, too, must be based on consensus [28] rather than any “gold standard”). It is likely that our questionnaire will be capable of *approximating* “possible” neuropathic pain in large samples [30].

### Implications of the findings

It is anomalous that, while there was strong consensus that “refractory” and “pharmacoresistant” neuropathic pain are different from each other, there was consensus (84%) on the definition of “refractory” neuropathic pain. Although the *a priori* level of 70% consensus was not achieved for the importance of trying non-pharmacological treatments, agreement approached this (63%), and free text comments supported it, and these treatments are therefore likely to represent the main difference between these two concepts. Psychological treatments and pain management programme, for example, may be effective in neuropathic pain [34,35]. Although “pharmacoresistant” neuropathic pain is not itself a consensus term, as our “refractory” neuropathic pain is, it is based on international consensus of a treatment paradigm of efficacious drugs [14,36].

These and other consensuses on pharmacological treatment, [36-39] provide an excellent basis on which clinicians can determine the order and nature of drugs to be used in neuropathic pain. Importantly, they also provide a framework for inquiring about treatment trials in epidemiology research. Whereas most of these focus on treatment in specialist settings, the recent guidelines from the National Institute for Health and Clinical Excellence (NICE) in the UK [39] focused on the non-specialist setting. The recommendations in the NICE guideline are broadly consistent with determining refractoriness at the point of specialist referral (that is, after a trial of three or four effective drugs).

It is important, as a far as possible, to use standard definitions and tools in research, including epidemiology. Good epidemiology provides a foundation basis for identifying resource, educational, treatment and prevention strategies, and for the subsequent evaluation of these. The use of standard tools allows comparison between different geographical areas, clinical and demographic subgroups, and periods of time. This study has allowed progress towards this for research on (refractory) neuropathic pain. Further work is now required to validate our proposed criteria in a general population study, and to review results of such a study in comparison with existing data, based on the range of instruments currently available. Our proposals represent a distilled compromise between the best features of existing instruments, with consensus from experts internationally.

## Competing interests

The authors declare that they have no competing interests.

## Authors’ contributions

All lead investigators (BHS, NT, MB, MS, KD) contributed to the conception and design of this study. Data collection and analysis were conducted by JF and NT. All authors were involved in the interpretation of data. BHS lead the writing of this manuscript and produced the first complete draft. All authors contributed to, and commented on all drafts, and approved the final manuscript.

## Pre-publication history

The pre-publication history for this paper can be accessed here:

http://www.biomedcentral.com/1471-2377/12/29/prepub
